# GeometryFormer: Semi-Convolutional Transformer Integrated with Geometric Perception for Depth Completion in Autonomous Driving Scenes

**DOI:** 10.3390/s24248066

**Published:** 2024-12-18

**Authors:** Siyuan Su, Jian Wu

**Affiliations:** National Key Laboratory of Automotive Chassis Integration and Bionics, Jilin University, Changchun 130025, China; wujian@jlu.edu.cn

**Keywords:** depth completion, deep learning, convolutional neural network, vision transformer, geometric perception, depth refinement

## Abstract

Depth completion is widely employed in Simultaneous Localization and Mapping (SLAM) and Structure from Motion (SfM), which are of great significance to the development of autonomous driving. Recently, the methods based on the fusion of vision transformer (ViT) and convolution have brought the accuracy to a new level. However, there are still two shortcomings that need to be solved. On the one hand, for the poor performance of ViT in details, this paper proposes a semi-convolutional vision transformer to optimize local continuity and designs a geometric perception module to learn the positional correlation and geometric features of sparse points in three-dimensional space to perceive the geometric structures in depth maps for optimizing the recovery of edges and transparent areas. On the other hand, previous methods implement single-stage fusion to directly concatenate or add the outputs of ViT and convolution, resulting in incomplete fusion of the two, especially in complex outdoor scenes, which will generate lots of outliers and ripples. This paper proposes a novel double-stage fusion strategy, applying learnable confidence after self-attention to flexibly learn the weight of local features. Our network achieves state-of-the-art (SoTA) performance with the NYU-Depth-v2 Dataset and the KITTI Depth Completion Dataset. It is worth mentioning that the root mean square error (RMSE) of our model on the NYU-Depth-v2 Dataset is 87.9 mm, which is currently the best among all algorithms. At the end of the article, we also verified the generalization ability in real road scenes.

## 1. Introduction

In recent years, with the development of artificial intelligence and deep learning, obtaining accurate and reliable depth maps is of great significance for autonomous vehicles. Currently, depth sensors, such as LiDAR, can provide reliable and accurate depth information and are therefore increasingly used. However, the problem is that the depth points obtained by the depth sensor are highly sparse, irregularly distributed, and there is noise near the edges of the object. These issues make depth completion a highly challenging task.

For this sparse to dense task, traditional methods are manual methods [1,2,3], which globally limit the output depth values and are usually difficult to obtain accurate results. Later, with the rise of deep learning, predicting depth maps based on neural networks became the most popular method, and related research also explained its huge advantages. Deep learning methods [4,5,6,7,8,9,10,11,12,13,14,15,16] recover depth values by fusing the global features of RGB images and the local features of sparse points. Global features can guide the extraction of local features and usually play a more important role [10]. Therefore, fully extracting global features has become a crucial factor affecting the final performance. Most previous algorithms employed widely used convolutional layers or graph propagation models to learn long-distance relationships; however, both convolutional layers and graph models focus more on local regions [12], such as 3 × 3 kernel size for convolution and kNN-based neighborhood for graph models. Therefore, vision transformer (ViT), which is highly favored for its excellent ability to fit long-range dependencies, has become the best choice for extracting global features [17]. Especially for outdoor autonomous driving scenarios, the cameras used usually have a very wide field of view, a large shooting range, and a long distance, so there are more scenes in the RGB images, and the semantic features are more complex. In this way, it is extremely difficult to establish long-range dependencies using only 3 × 3 convolutional layers, and the advantages of Transformer become increasingly obvious.

CompletionFormer [18] is a proposed a method of connecting ViT and convolutional layers in parallel as the basic unit forming the backbone, which is currently the most advanced method. However, there are still two serious flaws. On the one hand, the local information on the grid lines will be lost during tokenization [19]. To preserve some local features for the transformer as much as possible, we use a convolutional layer to predict the self-attention matrix to preserve the details on the grid lines and add depth-wise convolution to the feed-forward module to improve the local continuity. In this way, we convert the original ViT into a semi-convolutional style transformer, which has not been used for depth completion.

**Figure 1:**
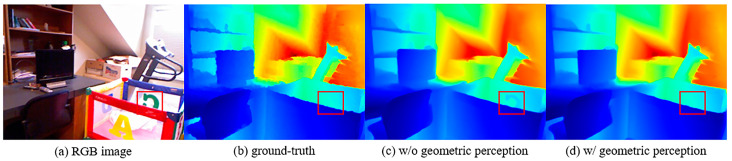
The depth values in the transparent plane with significant semantic changes are recovered incorrectly without geometric perception.

On the other hand, depth completion relies heavily on color images to extract color-dominant information such as object boundaries to complete sparse mapping [8]. When there are planes with irregular pixel values in the color image, it will lead to incorrect depth value prediction. For example, in a transparent plane area where the semantic information changes drastically, as shown in Figure 1, the part inside the box has obvious edges, but it is a plane. The previous transformer algorithm [18] mistakenly assumes that the depth of the box region changes dramatically when extracting RGB features, resulting in significant errors in the final depth map. Therefore, we designed a geometry perception module to help the transformer perceive the three-dimensional geometric structure. Based on the principle of triangulation [12], we mapped the coordinates of sparse points from the pixel coordinate system to the camera coordinate system and employed deformable convolutional network (DCN) [20], which is robust to non-rigid data, to learn the geometric features of sparse points in three-dimensional space. As a result, the distribution of edges and planes in the entire depth map could be inferred, which also helps guide the recovery of the edges and details in final depth map. This semi-convolutional transformer that integrates geometric features is named Geometry Transformer (GeT).

In addition, as described in CompletionFormer [18], transformer performs poorly on local feature details, and models based solely on transformers, such as GuideFormer [15], still have performance and efficiency gaps compared to developed CNN models. Thus, for the extraction of local features, we designed a multi-level local context module. This module consists of three convolutional neural network (CNN) branches but corresponding to different receptive fields to enhance the ability of obtaining local features. After the local context module, we use a parallel connection structure of channel and spatial attention to narrow the semantic gap between transformer and convolution. Although CBAM [21] is a method proposed for connecting the two in series, through ablation studies we found that the parallel approach can achieve better results than the series approach.

Finally, for integrating transformer and convolution more thoroughly, we fuse the output of the local context module with GeT in a double-stage form after the self-attention and feed-forward module, respectively. In this way, global and local information will flow throughout the block, information fusion and exchange are occurring inside it at any time, and we take the entire Geometry Transformer and Local Context (GTLC) block as the basic unit that constitutes the U-Net backbone and name it GeometryFormer. In summary, our contributions are as follows:

1. To prevent the loss of local information on the grid line during tokenization and maintain local continuity, we propose semi-convolutional style vision transformer, which utilizes a convolution layer to calculate the self-attention matrices and add depth-wise convolution to the feed-forward module.
2. For the accurate recovery of edges and details, we propose a geometric perception module that learns the distribution of geometric structures such as edges and planes by encoding the three-dimensional geometric features of sparse points and integrates them with transformer to improve network performance.
3. We designed a better double-stage fusion of convolution and transformer than the previous single-stage method; our GTLC block is employed as the basic unit that constitutes the whole U-Net backbone. Our model achieves SoTA performance on public datasets and still has excellent performance even when the depth data are extremely sparse.

In this paper, we propose a depth completion method based on the fusion of transformer and convolution. We introduce the related research of each component in Section 2 and various components and workflows in detail in Section 3. Section 4 and Section 5 are the experimental parts. Section 4 shows the comparative experiment with other SoTA algorithms and the ablation studies on the NYU-Depth-v2 and KITTI Datasets. Section 5 is the experiment conducted in real street scenes to evaluate the generalization ability of our algorithm.

## 2. Related Research

According to the different input data, depth completion methods can be divided into two categories: depth-only and RGB-guided. Depth-only methods [22,23,24,25,26] only take sparse depth maps as input and have poor performance. After Ma et al. [4] first proposed the RGB-guided method in 2019, depth-only methods have rarely been studied; therefore, this paper only discusses relevant research on RGB-guided methods. RGB-guided [4,5,6,7,8,9,10,11,12,13] methods take monocular RGB images and sparse depth points as input, and they extract the rich semantic information in the RGB image to guide the depth points for completion, so the effectiveness and accuracy are often better than depth-only methods. Due to the existence of two different modalities, previous researchers have explored a variety of deep learning methods to study how to fuse multi-modality features more efficiently. Representative methods include guidance map [4,5,6,7], surface normal [8,9], uncertainty [10,11], symmetric gated fusion [12], and dynamic convolution kernel [13], etc. Gansbeke et al. [10] built a two-branch network to extract global and local features; the global depth map output by global network is used to guide the local network. Then, uncertainty is employed to fuse the results of two branches for obtaining the final depth map; Qiu et al. [8] generated the surface normal based on RGB images, and the geometric clues of normal maps were introduced to guide the prediction of dense depth maps; Tang et al. [13] proposed dynamic convolution kernels to learn the weights of RGB network and deep network flexibly; Zhao et al. [12] constructed a graph propagation network to capture spatial information and utilize attention mechanisms to adaptively fit contextual information during the propagation process.

Although various multi-modality fusion methods have been proposed, due to their limited receptive field, the global perception ability of CNN-based methods is poor. Especially when the depth points are sparse, it is difficult to achieve satisfactory results. Due to stronger ability to fit long-range dependencies, ViT is more suitable for encoding global features than CNNs. ViT has demonstrated outstanding performance in many 2D computer vision tasks [27,28,29,30], and various ViT backbones [31,32,33,34,35,36] have also been used in 3D computer vision tasks. PVT [31] is a proposed pyramid ViT backbone that overcomes the difficulty of porting transformer to various dense prediction tasks. PVT-v2 [32] is a further proposed linear spatial reduction (LSR) layer with overlapping patch embedding, which reduces the computational complexity and achieves better performance. In addition to the transformer for providing global features, convolutional layers that encode local features of sparse depth points are still necessary, so this paper integrates transformer and CNNs to extract multi-modal features. Inspired by [31,32], we also constructed the network in the form of a multi-scale pyramid and used LSR to reduce the computational complexity.

However, ViT has a critical shortcoming, which is that the feature map needs to be tokenized because it focuses on modeling long-range dependencies. Such an operation will lose local information on the grid lines, resulting in damage to local continuity. For this issue, Lin et al. proposed a plug-and-play, fully convolutional style ConvFormer [19] for medical image segmentation, which consists of pooling, CNN-style self-attention, and convolutional feed-forward network, corresponding to the tokenization, self-attention, and feed-forward network of ViT, respectively; it optimizes the problems of attention collapse and loss of local information on small-scale training data. However, our method is different from theirs, as we only adopt a convolutional layer to predict the self-attention matrices, but feature maps are still tokenized when calculating the self-attention because we have found that CNN-style self-attention is difficult for achieving desired results in depth completion through ablation experiments. After the above operations, ViT is converted into a semi-convolutional transformer.

Then, depth completion is a 3D computer vision task, where three-dimensional geometric features play a crucial role. Previous studies [8,12,37] have explored various ways of obtaining geometric information. Zhao et al. [12] learned the geometric structure of the scene in the camera coordinate system and found that more multi-modality information can be obtained and observed in the three-dimensional coordinate system, and the root mean square error (RMSE) is also better than the two-dimensional coordinate system; Qiu et al. [8] introduced geometric information by estimating surface normal directly from the original input, which significantly improves depth prediction at occlusions; Su et al. [37] utilized gradient and normal loss functions to supervise training, which has excellent performance in recovering edges and continuously changing details. However, we found that the methods in [8,12] require significant computing power. The loss functions in [37] could improve visual effects to some extent, but they have almost no effect on performance indicators such as RMSE. Our aim of implementing geometric perception is to determine whether the current position is a plane or edge based on the positional correlation between local sparse points for recovering contours and details accurately. We map the position of sparse points from the pixel coordinate system to the camera’s coordinate system according to the principle of triangulation, based on camera intrinsic parameters, thereby obtaining a 3D position map. Then, a DCN [20] is implemented for encoding the 3D geometric information, which is added to the position embedding of the transformer to jointly monitor the absolute position of the depth points. Since only convolution operations are used, our method of obtaining geometric features is simpler and more convenient than previous methods [8,12].

Finally, there is a substantial semantic gap between transformer and CNNs, and promoting the integration of the two is an urgent problem to be solved. MonoViT [16] is a proposed Joint CNN and Transformer Layer, which consists of Multi-Path Transformer Block and depth-wise convolution. This module combines ViT’s global inference with the flexibility of self-supervised monocular depth estimation, enabling the model to infer both locally and globally, producing finer details and more accurate depth predictions. CompletionFormer [18] connects ViT and convolutional attention layer in parallel as the basic unit to construct the network in a multi-scale style, which is inspired by [32]; the backbone achieves higher accuracy and lower computational overhead than other general feature backbones [33,34,35,36]. However, we found that [16,18] simply concatenate the outputs of the two only once, which is difficult to achieve complete fusion, resulting in many outliers in the final depth map of complex outdoor scenes. To solve this problem, we proposed a double-stage fusion approach, which integrates the features of the convolutional layer after the self-attention module and the feed-forward module. This double-stage method can achieve a more thorough fusion effect than single stage, greatly improving the performance of the network.

In summary, unlike previous CNN-based methods, we integrated transformer and CNNs to extract multi-modal features. For the shortcomings of the transformer, we improved it into a semi-convolutional transformer and proposed a simple and convenient way to obtain geometric features so that the transformer has three-dimensional perception capabilities. For the fusion of transformer and CNNs, we proposed a more effective double-stage fusion method, which is more efficient than the previous naive single-stage method.

## 3. Method

**Figure 2:**
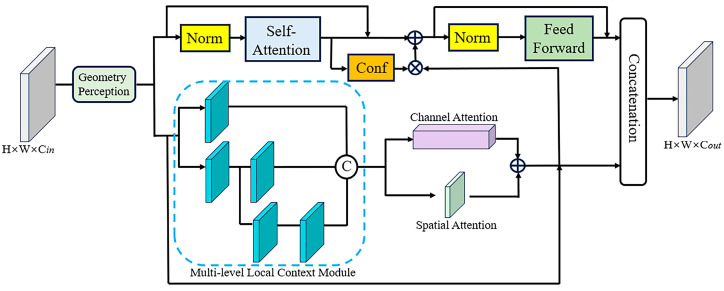
Geometry Transformer and Local Context Block. We connect convolution and transformer in parallel and fuse the local features extracted by the convolutional layer after the self-attention and feed-forward modules.

The GTLC module is employed as the basic unit for constructing a U-Net backbone. It is shown in Figure 2; we will introduce its various components in Section 3.1, Section 3.2, Section 3.3 and Section 3.4. Among them, the semi-convolutional transformer extracts global features, the geometry perception module provides it with three-dimensional geometric information, and the multi-level local context module encodes local features; the acquired global and local features are fused using a double-stage fusion strategy. Then, in Section 3.5 and Section 3.6, we specifically describe the composition of the encoder and decoder in detail, respectively, and finally in Section 3.7, we introduce the refinement strategy and loss function.

### 3.1. Semi-Convolutional Transformer

**Figure 3:**
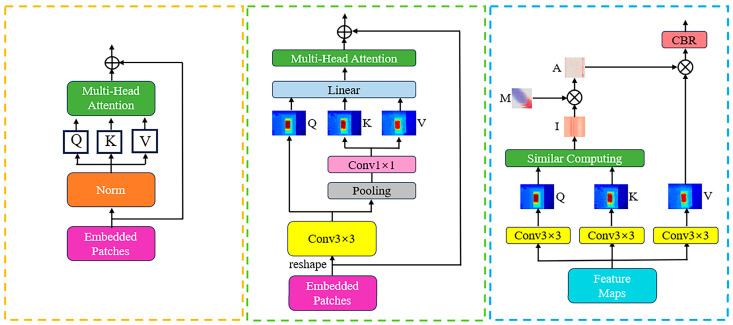
Comparison of our self-attention with ViT and ConvFormer. The left is self-attention of ViT, which directly generates the self-attention matrix after tokenization; the middle is our semi-convolution self-attention, which predicts the self-attention matrix by convolution layer; the right is pure convolution self-attention of ConvFormer, which generates dynamic convolution kernels to build long-range dependency.

After tokenization and layer normalization, we reshape the vectors into feature maps, and a convolutional layer is implemented to predict the Q, K, and V matrices to perform feature fusion and exchange for preserving local information to a certain extent. To reduce the amount of calculation, we adopt the linear spatial reduction (LSR) layer proposed by PVT-v2 [32] to reduce the dimension when calculating K and V. Figure 3 shows, from left to right, self-attention of original ViT, our semi-convolutional self-attention, and pure convolutional self-attention of ConvFormer. We also try to use the convolutional self-attention of Lin et al. [19], that is, introduce a learnable Gaussian distance map to dynamically determine the size of the convolution kernel to build adaptive long-range dependency, thereby obtaining the global receptive field. However, our experimental results show that the accuracy and efficiency of this are not as good as ViT’s token-based self-attention mechanism in depth completion. Therefore, we still apply token-based self-attention and tokenize the feature maps after Linear SR, which can be formulated as follows:

(1) SR(x)=Norm(Linear(AvgPool(x,Pi))).

where $x\in R^{Hi\times Wi\times Ci}$ is the input feature map, *Pi* is pooling size, and Linear SR utilizes average pooling to reduce the input size from *Hi* × *Wi* to *Pi* × *Pi*. Then, Linear and Norm refer to tokenization and layer normalization, respectively. Similar to the original ViT, our attention operation is calculated as follows:

(2) $$\mathrm{Attention}(Q,K,V)=\mathrm{Softmax}(\frac{QK^{T}}{\sqrt{d_{head}}})V.$$

where Q, K, and V are Query, Key, and Value, respectively; *d_head_* is the dimension of each head; then, the attention operation of each head can be expressed as follows:

(3) $${\mathrm{head}}_{j}=\mathrm{Attention}(QW_{j}^{Q},SR(K)W_{j}^{K},SR(V)W_{j}^{V})$$

The final output is the following:

(4) $$\mathrm{Attn}=\mathrm{Concat}({\mathrm{head}}_{0},\ldots ,{\mathrm{head}}_{N_{i}})W^{O}$$

where Concat(·) is the concatenation operation, and $W_{j}^{Q},W_{j}^{K},W_{j}^{V}\in R^{C_{i}\times d_{head}}$ are linear projection parameters. *N_i_* is the head number of self-attention on stage *i*.

### 3.2. Geometric Perception Module

**Figure 4:**
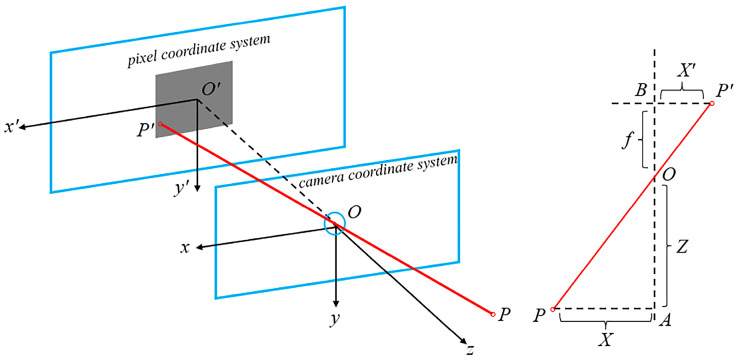
The left image is a projection diagram. The point *P* in the camera coordinate system is projected onto the pixel plane as *P′*, while the right image shows the geometric relationship of triangulation; ΔAOP is similar to ΔBOP′.

We map the coordinates of sparse points from the pixel plane to the camera coordinate system based on the principle of triangulation, as shown in Figure 4 and Formulas (5) and (6), where (*u*, *v*) and (*u*_0_, *v*_0_) are the coordinates of sparse points and optical centers in the pixel plane, respectively; *f_x_* and *f_y_* are the camera focal lengths. According to Formula (6), the coordinates of sparse points in the X and Y directions in the camera coordinate system can be calculated. And D is the depth value, which happens to be the Z-axis coordinate in the camera coordinate system. Our main goal is to infer the distribution of geometric structures such as planes and edges by learning the positional relationships and geometric features of sparse points in three-dimensional space, thereby supervising the edges and contours of objects in dense depth maps and solving the problem of unclear or offset edges.

(5) Z=D

(6) $$X=\frac{(u-u_{0})Z}{f_{x}},Y=\frac{(v-v_{0})Z}{f_{y}}$$

**Figure 5:**
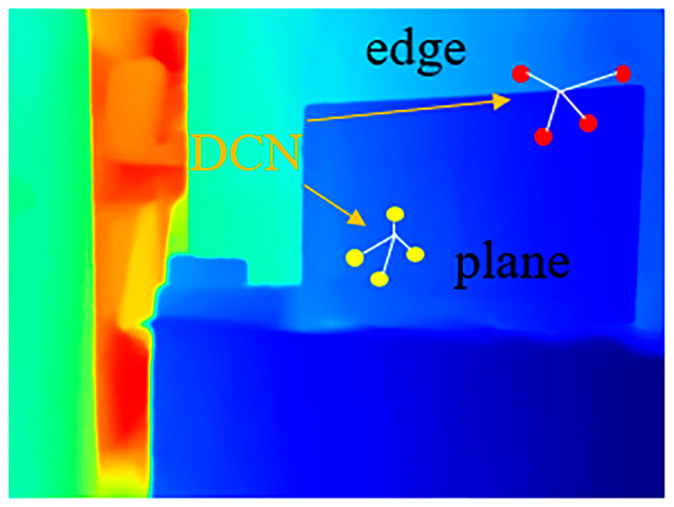
Different geometric features at edges and planes.

CoordConv [38] uses conventional convolution to encode pixel coordinates, but in this paper, the coordinates of sparse points are irregular, and conventional convolution is limited by a limited receptive field, making it difficult to perform well. Therefore, we introduce a more robust deformable convolutional network (DCN) [20] to encode the three-dimensional position map of sparse points for non-rigid data. As shown in Figure 5, if the depth value encoded by DCN [20] changes dramatically, it can be perceived as an edge (red points); otherwise, it is a plane (yellow points). By encoding the geometric features of sparse points at various local positions by DCN, the distribution of edges and planes in the entire depth map can be inferred.

**Figure 6:**
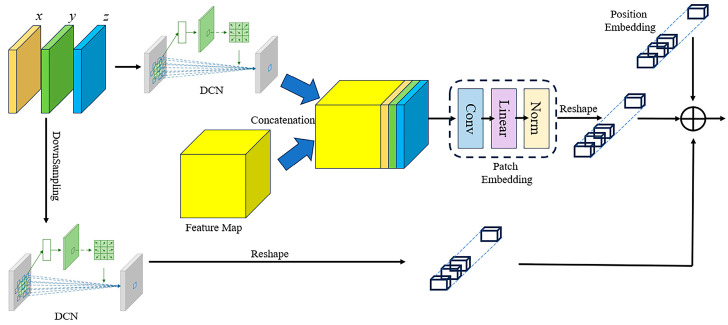
Geometric Perception. When the input data are in the form of feature maps, the three-dimensional coordinate map is concatenated to the input. After the feature map is tokenized, the coordinate map is also reshaped into a vector form and added to the tokens.

In addition, we employ sparse pooling to downsample the three-dimensional position map and transfer it to stages of various corresponding scales. In each stage, the geometric information is constructed into two branches. The first branch is preprocessed by deformable convolution [20] and connected to the input feature maps of the layer. The second branch is tokenized and added with position embedding, because we believe that geometric information and position embedding complement each other. Position embedding can supervise the position of the patches, while geometric information can supervise the position of depth points. The overall schematic diagram of the geometric perception module is shown in Figure 6.

### 3.3. Multi-Level Local Context Module

Inspired by the SSH [39] structure, which is popular in face detection, we employ it in our network to enhance the modeling ability of local contexts because it is scale-invariant by design and able to collect context information with different scales in one forward pass. It can extract features of different receptive fields during propagation and is fast and lightweight. The three output branches correspond to receptive fields 3, 5, and 7, and relevant features will flow to the corresponding branches adaptively. Finally, to make up for the huge semantic gap with the transformer, we add parallel channels and spatial attention after the multi-level local context module.

### 3.4. Fusion Strategy of Transformer and Convolution

There is a large semantic gap between transformer and convolution. Perfectly integrating the two is a crucial issue for depth completion. The multi-level context module redistributes weights after parallel channels and spatial attention, and the semantic gap between the two is reduced. However, it is not suitable to adopt crude direct concatenation at this time, so a weight is assigned to the convolutional layers for weighted fusion based on the self-attention module, which adaptively learns the amount of local information required by transformer. The self-attention module is followed by the feed-forward module, which performs non-linear mapping and feature extraction on the input feature vectors, and we apply depth-wise convolution to improve the continuity of local features, further reducing the semantic gap between the two branches, so direct concatenation is performed. After two stages of fusion, the semantic information of the transformer and convolution are perfectly fused together.

### 3.5. Encoder

There are 5 stages in our encoder, and the smallest feature map is downsampled 32 times compared to the original input size. Since the feature maps of the first stage are too large, large numbers of tokens will be generated by transformer, which will greatly increase the amount of calculation and inference time. Therefore, inspired by CompletionFormer [18], we also adopt the BasicBlocks of resnet34 [40] in the first stage, and take our GTLC module for the remaining 4 stages. For each stage, it consists of a patch embedding module and N*i* repeated GTLC modules. We list the N*i* of the 4 stages in Table 1 and divide the model into 3 different scales, and the channels for them are 64, 128, 320, and 512. The patch embedding module is a 2 × 2 convolution layer with stride 2 to halve the resolution for the next stage.

### 3.6. Decoder

In Decoder, common upsampling methods are not introduced, such as deconvolution or interpolation. Irrelevant information will be introduced by deconvolution when performing padding, which will interfere with the recovery of depth values. The interpolation methods that insert virtual values between two pixels will also leave traces of artificial synthesis. These problems will negatively influence the recovery of the final depth map. We first use a pointwise convolution with a stride of 1 and kernel size of 1 × 1 to generate the required number of channels, and then adopt pixel-shuffle to double the feature map scale; because pixel-shuffle utilizes its own pixels when upsampling, no other interference values are introduced. Finally, the output of each encoder will be concatenated with the input of the decoder of the same size through a skip structure to complete the exchange and fusion of features.

### 3.7. Depth Refinement and Loss Function

Non-local methods such as DSPN [41], DySPN [42], and NLSPN [43] can often achieve better refinement than fixed-local methods such as CSPN [44] and CSPN++ [45]. Recently, Liu et al. [46] proposed GraphCSPN based on graph convolution, which extends the propagation process from two dimensions to three dimensions. We conducted ablation studies on DySPN [42], NLSPN [43], and GraphCSPN [46] and found that NLSPN [43] is more suitable for our backbone and can achieve the lowest RMSE.

In addition, although the original NLSPN can flexibly select pixels in the neighborhood, we believe that when the kernel size is 3, the neighborhood range is not large enough. If the convolution kernel size is increased to 5 or 7, the required calculations and memory usage will be greatly increased. Therefore, we adopted three layers of NLSPN and constructed them in the form of a dilation pyramid. The kernel size of each layer remains unchanged at 3, but the dilation rate increases sequentially, with values of 1, 2, and 3. In this way, the receptive fields can reach 3, 5, and 7, respectively. Compared with a single dilation rate, the performance and accuracy are further improved.

The rough depth map is $P_{init}$; $P^{t}=(p_{u,v}^{d,t})\in R^{H\times W}$ represents the depth map obtained by the *t*-th iteration propagation, and *d* represents the dilation rate of spatial propagation. $p_{u,v}^{d,t}$ represents the depth value at (*u*, *v*), *H* and *W* represent the height and width of the depth map *P^t^*, respectively. If there are *T* iterations of propagation under each dilation rate, the final depth value of each pixel can be described as follows:

(7) $$p_{u,v}=\sum_{d\in (1,2,3)}\sum_{t\in T}\left(w_{u,v}(0,0)p_{u,v}^{d,t-1}+\sum_{(i,j)\in N_{u,v},i\neq 0,j\neq 0}w_{u,v}(i,j)p_{i,j}^{d,t-1}\right)$$

In this way, we improve the refinement accuracy by gradually expanding the neighborhood range, and each iteration only extracts the affinity of 8 neighborhood pixels, achieving a compromise between accuracy and calculation consumption. After 3*T* iterations, we obtain the final refined depth map *P_out_*.

As for the loss function, similar to most previous SoTA algorithms, we also adopt L1 and L2 losses to supervise the training of the network as follows:

(8) $$L(\overset{⌢}{P},GT)=\frac{1}{\left|K\right|}\sum_{k\in K}\left(\left|{\overset{⌢}{P}}_{k}-GT_{k}\right|+{\left|{\overset{⌢}{P}}_{k}-GT_{k}\right|}^{2}\right)$$

where $\overset{⌢}{P}=P_{out}$; *K* is the number of effective depth points involved in loss calculation in ground truth *GT*.

## 4. Experiments

### 4.1. Datasets

NYUv2 Dataset [47]: It contains 464 indoor scenes captured by Microsoft Kinect [48], with corresponding RGB images and dense depth maps for each frame. Similar to the previous SoTA methods, we use approximately 50 K images as a training set and evaluate on a test set containing 654 images. The original sizes of the training set and test set are both 640 × 480. For training, we first downsample each frame to 320 × 240 during data preprocessing, then perform random flipping, rotation, scaling and brightness adjustment, and finally perform center cropping to 304 × 228. For evaluation, only downsampling and center cropping were performed, and evaluation indicators were calculated on the size of 304 × 228. Evaluation indicators include root mean square error (RMSE), mean absolute error (MAE), and mean absolute relative error (REL).

KITTI Depth Completion Dataset [22]: It is a large dataset of street scenes captured on a moving car, mainly used for autonomous driving. Each frame has a color image, sparse depth map, and ground-truth depth map. The sparse depth map was acquired by Velodyne HDL-64E, and the ground truth was generated by collecting 11 consecutive time frames of the LiDAR scan into one, resulting in approximately 30% annotated pixels. It contains 86 K training data, 1000 evaluation data, and 1000 test data without ground truth. Like NYUv2, we also randomly flip, rotate, and scale the original frame, and finally make a 256 × 1216 bottom crop because there is no LiDAR return at the top of the image. Evaluation indicators include root mean square error (RMSE), mean absolute error (MAE), root mean square error of the inverse depth (iRMSE), and mean absolute error of the inverse depth (iMAE).

The following formula shows the detailed definition of each indicator:

(9) $$RMSE=\sqrt{\frac{1}{m}\sum_{i=1}^{m}(y_{i}-y_{gt}{)}^{2}}\;MAE=\frac{1}{m}\sum_{i=1}^{m}\left|y_{i}-y_{gt}\right|$$

(10) $$iRMSE=\sqrt{\frac{1}{m}\sum_{i=1}^{m}(1/y_{i}-1/y_{gt}{)}^{2}}\;iMAE=\frac{1}{m}\sum_{i=1}^{m}\left|1/y_{i}-1/y_{gt}\right|$$

(11) $$REL=\frac{1}{m}\sum_{i=1}^{m}\left|(y_{i}-y_{gt})/y_{gt}\right|$$

### 4.2. Implementation Details

We trained and evaluated our model using an Intel Core i9 13900K CPU (Intel, USA) and two NVIDIA RTX 4090 GPUs (NVIDIA, USA), on Ubuntu 20.04 with Python 3.8 and PyTorch [49] 2.0.1. We used AdamW [50] as optimizer with an initial learning rate of 0.001, β1 = 0.9, β2 = 0.999, weight decay of 0.01. The batch size was set to 16 for NYUv2 and 3 for KITTI DC per GPU, and the initial learning rate was set to 0.001. We trained the model on the NYUv2 Dataset for 64 epochs, and the learning rate was reduced by half at epochs 32, 40, 48, and 60. For the KITTI DC Dataset, we trained the model for 80 epochs and halved the learning rate at epochs 30, 40, 50, 60, and 75. Note that most transformer models need to be pretrained on large datasets to achieve the desired performance, but our model was only trained on the two datasets without any additional data.

### 4.3. Comparison with SoTA Methods

We compared the performance indicators with other SoTA methods on the NYUv2 and KITTI DC Datasets, as shown in Table 2. For the NYUv2 Dataset, the indicators we compared are RMSE and MAE. For the KITTI DC Dataset, we compared the RMSE, MAE, iRMSE, and iMAE of the test dataset. We submitted our results on the KITTI Vision Benchmark Suite at the time of paper submission, and all our indicators are comparable to the latest SoTA methods.

**Figure 7:**
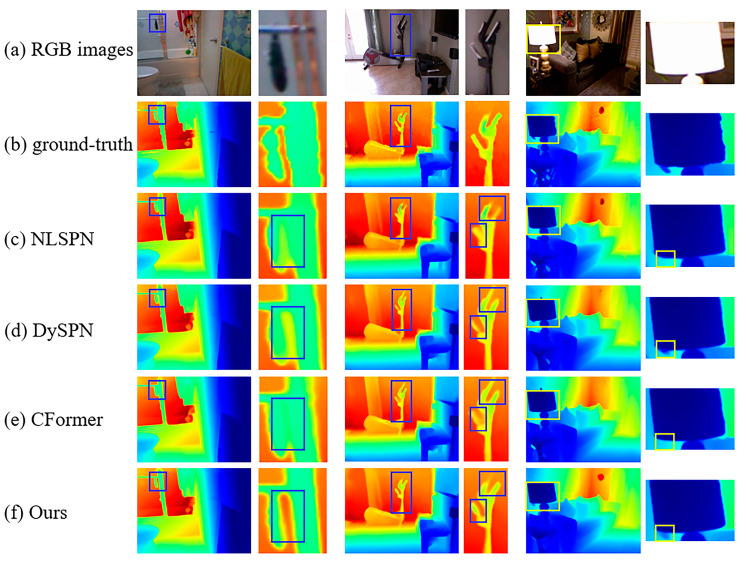
Qualitative results on NYUv2 Dataset. Comparisons of our method against SoTA methods.

**Figure 8:**
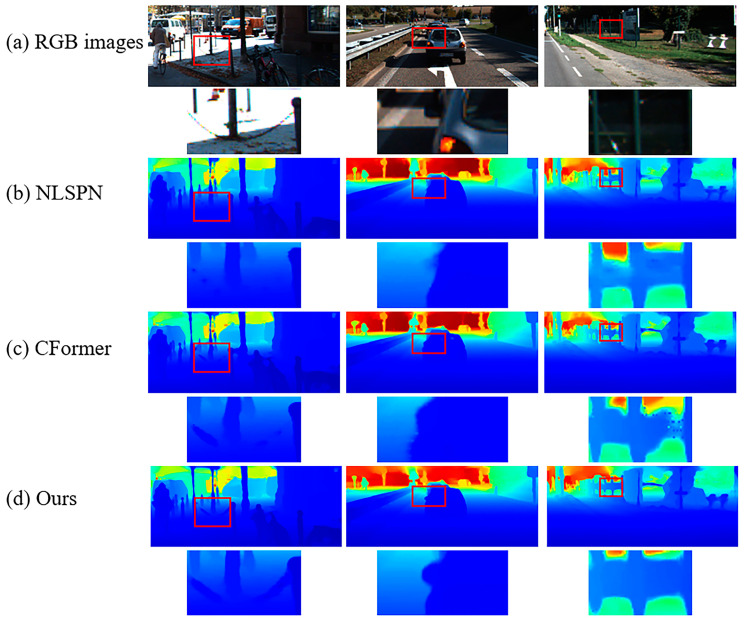
Qualitative results on KITTI DC test dataset. Comparisons of our method against SoTA methods.

Qualitative results for the NYUv2 and KITTI DC Datasets are provided in Figure 7 and Figure 8. For the NYUv2 Dataset, our model performs better for outlines and edges (e.g., there is no adhesion phenomenon in our results in the first row) and tiny structures (e.g., the handle of the treadmill is almost identical to the ground truth in the second row, and we recovered the leaves in the lower left corner of the desk lamp in the third row). For the KITTI DC test dataset, by integrating semi-convolution transformer and geometric perception, our method performs better on tiny objects (e.g., first and second row, we recovered the outline of the chain but the other two did not, and we recovered a clearer outline of the car rearview mirror than the others) and transparent glass (e.g., second row, our edges are more precise and, thanks to geometric perception, the depth values of transparent planes are smoother than the other algorithms.). The above detailed scenes often appear in autonomous driving data, and it is extremely difficult to recover depth values. Our model can handle these scenes well and meet the extremely high requirements of autonomous driving for depth maps.

**Figure 9:**
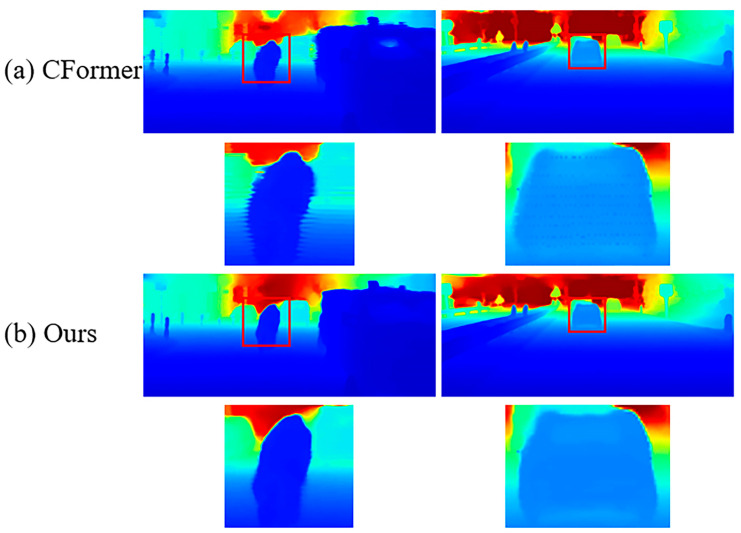
Visual effect comparison of single-stage and double-stage fusion.

In addition, we compared the impact of fusion strategies on the final depth map. In Figure 9, it can be seen that single-stage fusion will lead to a large number of outliers and ripples generated due to incomplete fusion; however, our double-stage fusion solves this problem and enhances the visual effect.

### 4.4. Ablation Studies and Analysis

The KITTI DC Dataset is large and takes about a week for training; thus, we evaluated our major components on the NYUv2 Dataset, requiring only one day for each training. Similar to the previous SoTA methods, we randomly sample 500 depth points from ground truth to generate sparse depth maps. Table 3 shows the ablation studies of the backbone, which is mainly divided into three parts. (1) and (2) compare the performance of ViT and semi-convolutional transformer, (3) to (6) compare the effects of various refinement methods on the backbone, and (8) to (17) are comparative experiments with other transformer algorithms. Table 4 shows the ablation studies of each component, which is also the optimization process of our backbone. We added or replaced one component per experiment and evaluated its performance.

*The effectiveness of backbone.* We compared with other transformer backbones, but did not include CMT [35], MPViT [36], Swin Transformer [33], etc., because related studies have been conducted in CompletionFormer [18]. For RMSE, our semi-convolutional backbone in small (12, 16) and base (17) scale outperforms the pure CNN-style ConvFormer [19] (8), the pure transformer algorithm (9), and the algorithms based on fusion of transformer and convolution (10). Compared with CFormer [18], our method can achieve better performance with lower computational complexity, while ConvFormer [19], which implements self-attention based on pure convolution, not only requires more computational resources but also has worse performance. It is worth mentioning that the difference between (1) and (2) is that only by changing ViT to semi-convolutional transformer, the RMSE drops from 88.9 to 88.1.

*Dilation Rate of SPN Refinement.* Our network (3) achieves excellent results after six iterations of propagation, but by increasing dilation rates to NLSPN to expand the range of neighborhood affinities, performance and accuracy are further improved. After increasing the dilation rate to 2 and 3, the RMSEs dropped to 88.0 (6) and 87.9 (7), respectively.

*Model Scale.* We divided the network into three scales based on the number of GTLC modules that make up the network, namely tiny (15), small (16), and base (17). Thanks to semi-convolution and geometric perception, our network consumes less computation and achieves better performance compared to the corresponding scale of CFormer [18]. In the following experiments, we selected the model in base scale with the dilation rates of {1,2,3} as our final structure.

*Geometric Perception.* Compared to (20), which has no geometry perception, the RMSE of (21) dropped from 88.8 mm to 88.4 mm, indicating that geometric perception module could infer the distribution of geometric structures such as planes and edges in depth maps based on the geometric features of sparse depths. Therefore, the details were optimized and the RMSE was reduced. It is worth mentioning that a similar 3D coordinate map is also used in PENet [6], but it is directly concatenated with the input feature map. We used DCN to further process the 3D map and convert it into a vector form and added it to tokens, making more thorough use of the 3D geometric features.

*Fusion Strategy.* In Table 4, 1 represents single-stage fusion, and 2 represents the double-stage fusion strategy we proposed. Experimental results show that double-stage fusion (22) is more efficient than single-stage fusion (21) and more conducive to taking advantage of the linkage between transformer and convolution.

*Cascade* vs. *Parallel Attention.* Although CBAM [21] is a method proposed for connecting spatial attention and channel attention in series, our experiments prove that connecting the two types of attention in parallel (20) can achieve better results than connecting them in series (19), and the RMSE decreases 0.2 mm.

*Decoder.* We adopted an upsampling method that combines pointwise convolution and pixel-shuffle in the decoder. The purpose is to avoid artificial traces and irrelevant information introduced by the naive upsampling method. It can be seen from Table 4 that the RMSE of our result (19) is much lower than widely used deconvolution (18).

### 4.5. Sparsity Level Analysis

To demonstrate the effectiveness and robustness of our model, we conducted performance evaluation under various sparsity levels on the NYUv2 and KITTI DC Datasets. For the NYUv2 Dataset, we randomly collected 0, 50, 200, and 500 from ground truth to simulate various levels of sparsity. For the KITTI DC Dataset, we followed [51] to sub-sample the raw LiDAR points in azimuth-elevation space into 1, 4, and 16 lines. We trained on 10,000 images provided by [18] and validated on the 1000 images of the selected validation dataset. To demonstrate the superiority of the semi-convolutional transformer, we compared it with ViT-based CFormer [18] and the state-of-the-art CNN method [43,53]. In addition, to evaluate the positive impact of geometric perception, Ours-ViT donates the ViT that we adopted in our network instead of semi-convolutional transformer, and the refined module is only propagated for six iterations with the dilation rate of 1 to facilitate a fair comparison with CFormer [18].

As can be seen from Table 5 and Table 6, Ours-ViT also performs well when the sparsity level is very high, indicating that the geometric perception module can adapt to autonomous driving scenes with particularly sparse depth values. After replacing ViT with semi-convolution transformer (Ours), the performance was further improved.

**Figure 10:**
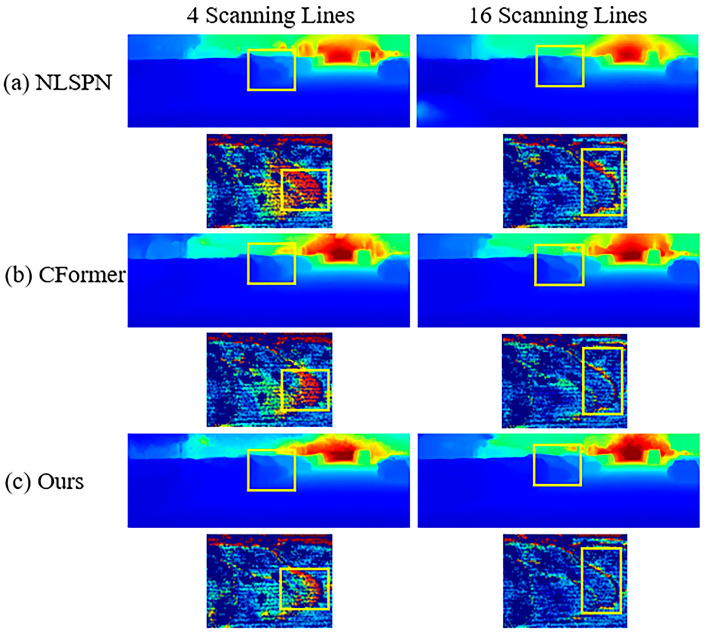
Qualitative results on KITTI DC selected validation dataset with 4 and 16 LiDAR scanning lines. Comparisons of our method against SoTA methods.

Figure 10 presents the qualitative results on the KITTI DC selected validation dataset with 4 and 16 LiDAR scanning lines. Our algorithm performs better in occlusions and edges (the area where multiple vehicles overlap in the first row). In summary, our method can still achieve fewer errors in the case of particularly sparse depth points with only four scanning lines.

## 5. Real Vehicle Test and Generalization Ability Verification

We ran our algorithm on the Baidu Apollo model car to verify the generalization ability in real road scenes. The sensors we used to collect data were a RoboSense 16-line LiDAR RS-Helios-16P and a ZED 2 camera. The on-board computer used was dSPACE AUTERA Autobox (dSPACE, USA), which has an Intel Xeon CPU with 12 cores and an NVIDIA RTX6000 GPU.

**Figure 11:**
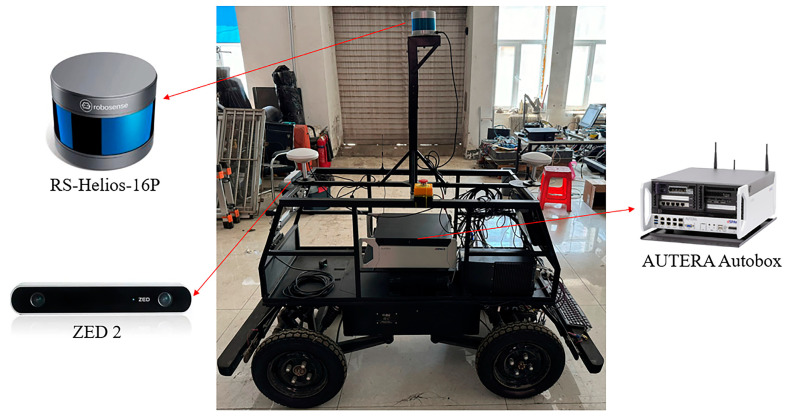
Our real-car experimental platform for collecting real road scenes data.

Our real vehicle experimental platform is shown in Figure 11.

**Figure 12:**
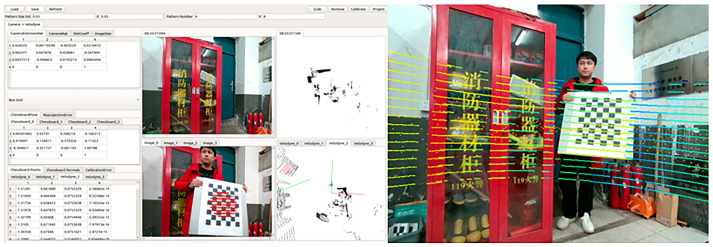
Our calibration process and results on Autoware.

**Figure 13:**
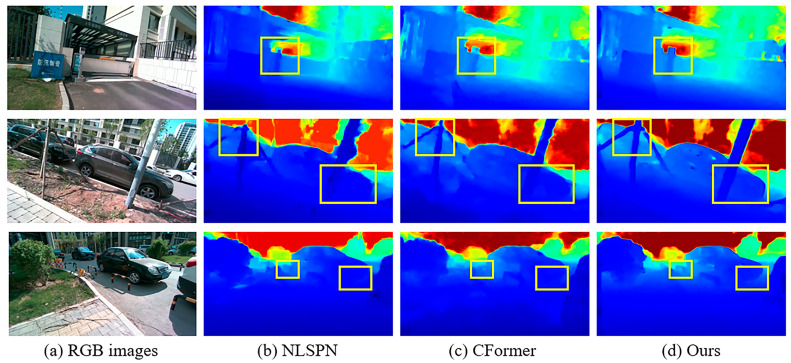
Comparison with SoTA method in real road scenes.

First, we used the autonomous driving framework Autoware to implement the joint calibration of the camera and LiDAR. The calibration process and results are shown in Figure 12. After that, we installed the Ubuntu 18.04 system on AUTERA Autobox to run our algorithm. The results obtained in real road scenes are shown in Figure 13. Our LiDAR has a small number of scan lines, and the real data are noisier than the data in the KITTI dataset. Even so, our results still outperform the state-of-the-art method based on fusion of CNN and transformer (CompletionFormer [18]) and CNN-based method (NLSPN [43]). It can be seen from Figure 13 that our method is better than others in recovering contours, details, and small objects.

## 6. Conclusions

This paper proposed a semi-convolution transformer that integrates three-dimensional geometric perception. Compared with the original vision transformer, both performance and accuracy have been significantly improved, and performance indicators have been improved while reducing computational complexity. After integrating geometric perception, the visual effect of the predicted dense depth map at edges and details is greatly improved, especially in transparent plane areas where semantic information changes drastically but depth is smooth, and it has better effects and efficiency than other SoTA algorithms even when the input is extremely sparse. In addition, the double-stage fusion strategy we proposed is more conducive to promoting the interaction of global information and local features than single-stage fusion and solves the problem of outliers and ripples caused by incomplete fusion. Our model not only achieves state-of-the-art results on both indoor and outdoor public benchmarks, but also outperforms other algorithms in generalization ability on real road scenes. Finally, the limitation of this paper is that tokenization and the calculation of self-attention will increase the computational complexity, so the inference time is longer than lightweight CNN-based algorithms. In future research, our goal is to explore faster self-attention calculation methods, and prune and quantize the model so that it can be applied to mass-produced autonomous driving systems. In addition, since the KITTI benchmark dataset does not have training data on abnormal weather, we have been developing algorithms such as low-light enhancement and deraining to cope with various weather conditions (the existing achievement paper is [54]), which will enable all-weather depth completion.

## Figures and Tables

**Table 1:** The scales of GeometryFormer. Layers donate the number of GCLC blocks in each stage. FLOPs are obtained when the input size is 640 × 480.

Scales	Layers	Params (M)	FLOPs (G)
Tiny	[2, 2, 2, 2]	56.8	334.0
Small	[2, 3, 3, 4]	94.1	354.3
Base	[3, 3, 4, 6]	130.5	374.7

**Table 2:** Comparison with SoTA methods on KITTI DC and NYUv2 Datasets.

Method	KITTI DC				NYUv2		Reference
	RMSE (mm)	MAE (mm)	iRMSE (1/km)	iMAE (1/km)	RMSE (m)	REL	
ACMNet [12]	732.99	206.80	2.08	0.90	0.105	0.015	TIP2021
GuideNet [13]	736.24	218.83	2.25	0.99	0.101	0.015	TIP2021
PENet [6]	730.08	210.55	2.17	0.94	-	-	ICRA2021
TWISE [51]	840.20	195.58	2.08	0.82	0.097	0.013	CVPR2021
PRNet [52]	867.12	204.68	2.17	0.85	0.104	0.014	CVPR2021
RigNet [5]	712.66	203.25	2.08	0.90	0.090	0.013	ECCV2022
GuideFormer [15]	721.48	207.76	2.14	0.97	-	-	CVPR2022
DySPN [42]	709.12	192.71	1.88	0.82	0.090	0.012	AAAI2022
Decomposition [53]	707.93	205.11	2.01	0.91	0.098	0.014	TCSVT2023
CFormer [18]	708.87	203.45	2.01	0.88	0.090	0.012	CVPR2023
Ours	702.64	190.86	1.92	0.82	0.0879	0.0114	-

**Table 3:** Ablation studies of the backbone type and the dilation of NLSPN refinement module on NYUv2 Dataset. FLOPs are obtained when the input size is 640 × 480.

	GeometryFormer	RMSE (mm)	MAE (mm)		Params. (M)	FLOPs (G)
(1)	origin ViT	88.9	34.6		125.8	370.4
(2)	w/semi-convolution	88.1	34.3		130.5	374.7
	Dilation Rates	Refinement Method	RMSE (mm)	MAE (mm)	Params. (M)	FLOPs (G)
(3)	-	DySPN	89.3	35.2	121.4	394.5
(4)	-	GraphCSPN	90.2	35.8	147.5	459.7
(5)	{1}	NLSPN	88.1	34.3	130.5	374.7
(6)	{1, 2}	NLSPN	88.0	34.3	130.5	379.4
(7)	{1, 2, 3}	NLSPN	87.9	34.1	130.5	384.2
	Backbone Type	Refinement Iterations	RMSE (mm)	MAE (mm)	Params. (M)	FLOPs (G)
(8)	ConvFormer	18	92.8	36.6	101.5	375.5
(9)	PVTv2-Base	18	91.2	35.4	126.7	446.8
(10)	CFormer-Small	18	90.1	35.2	82.6	439.1
(11)	Ours-Small	18	89.5	35.0	94.1	363.8
(12)	CFormer-Tiny	6	90.9	35.3	45.8	389.4
(13)	CFormer-Small	6	90.0	35.0	82.6	429.6
(14)	CFormer-Base	6	90.1	35.1	146.7	499.6
(15)	Ours-Tiny	6	90.2	35.0	56.8	334.0
(16)	Ours-Small	6	89.3	34.8	94.1	354.3
(17)	Ours-Base	6	88.1	34.3	130.5	374.7

**Table 4:** Ablation studies of components of our network on NYUv2 Dataset. The components include geometric perception module, fusion strategy, convolutional attention mechanism, and upsampling strategy in decoder.

	Geometric Perception	Fusion Strategy	Attention	Decoder	RMSE (mm)	MAE (mm)
(18)	×	1	Cascade	Deconvolution	89.4	34.8
(19)	×	1	Cascade	Pixel-shuffle	89.1	34.7
(20)	×	1	Parallel	Pixel-shuffle	88.8	34.6
(21)	√	1	Parallel	Pixel-shuffle	88.4	34.4
(22)	√	2	Parallel	Pixel-shuffle	88.1	34.3

**Table 5:** Sparisity studies on NYUv2 Dataset. We compare with SoTA methods on 0, 50, 200, and 500 samples.

Method		RMSE (m)				
		NLSPN	DySPN	CFormer	Ours-ViT	Ours
Sample Number	0	0.562	0.521	0.490	0.482	0.480
	50	0.223	0.203	0.208	0.201	0.198
	200	0.129	0.126	0.127	0.123	0.121
	500	0.092	0.090	0.090	0.0889	0.0879

**Table 6:** Sparsity studies on KITTI DC Dataset. We compare with SoTA methods on scanning lines of 1, 4, 16, and 64.

Scanning Lines	Method	RMSE (mm)	MAE (mm)	iRMSE (1/km)	iMAE (1/km)
1	NLSPN	3507.7	1849.1	13.8	8.9
	DySPN	3625.5	1924.7	13.8	8.9
	CFormer	3250.2	1582.6	10.4	6.6
	Ours-ViT	3211.9	1574.6	10.4	6.5
	Ours	3189.4	1561.3	10.4	6.5
4	NLSPN	2293.1	831.3	7.0	3.4
	DySPN	2285.8	834.3	6.3	3.2
	CFormer	2150.0	740.1	5.4	2.6
	Ours-ViT	2117.6	736.1	5.4	2.5
	Ours	2098.8	733.6	5.3	2.5
16	NLSPN	1288.9	377.2	3.4	1.4
	DySPN	1274.8	366.4	3.2	1.3
	CFormer	1218.6	337.4	3.0	1.2
	Ours-ViT	1191.7	332.5	3.0	1.2
	Ours	1178.5	327.0	2.9	1.2
64	NLSPN	889.4	238.8	2.6	1.0
	DySPN	878.5	228.6	2.5	1.0
	CFormer	848.7	215.9	2.5	0.9
	Ours-ViT	827.3	210.4	2.4	0.9
	Ours	819.4	206.9	2.3	0.8

## Data Availability

The raw data supporting the conclusions of this article will be made available by the authors on request.
